# Case report: Metastatic myxoid liposarcoma arising from the right atrium extends as cardiac tamponade—A rare case of atrial oncology

**DOI:** 10.3389/fcvm.2022.1046436

**Published:** 2023-01-26

**Authors:** Muralidharan Thoddi Ramamurthy, Vinod Kumar Balakrishnan, Sini Sunny, Abinayaa Rajkumar, Sandhya Sundaram, Preetam Krishnamurthy, Nagendra Boopathy Senguttuvan, Arunan Murali, J. D. Roy Santhosham, Kalaiselvi Periandavan, Namakkal S. Rajasekaran

**Affiliations:** ^1^Department of Cardiology, Sri Ramachandra Medical University and Research Institute, Chennai, Tamil Nadu, India; ^2^Division of Molecular and Cellular Pathology, Department of Pathology, University of Alabama at Birmingham, Birmingham, AL, United States; ^3^Department of Medical Biochemistry, University of Madras, Taramani Campus, Chennai, Tamil Nadu, India; ^4^Department of Radiology, Sri Ramachandra Medical University and Research Institute, Chennai, Tamil Nadu, India; ^5^Division of Cardiovascular Medicine, Department of Medicine, University of Utah, Salt Lake City, UT, United States

**Keywords:** liposarcoma—diagnosis, cardiac tamponade, right atrium (RA), cardiac tumor diagnosis, FDG (18F-fluorodeoxyglucose)-PET/CT

## Abstract

The reported incidence of liposarcomas in ~2,000 cases annually results in about 30% of myxoid liposarcomas. Cardiac myoxid liposarcomas are very rare; their presentation could be cardiac tamponade, due to direct compression of the tumor and/or pericardial effusion. In this report, we describe a patient who presented with pericardial effusion secondary to myoxid liposarcomas from the right atrium, an extremely rare presentation of liposarcomas in the heart. We also present non-invasive imaging through echocardiography, CECT thorax and FDG PET scans, followed by a CT-guided mass biopsy. Histopathology of the right atrial mass demonstrated myxoid liposarcoma positive for the S100 tumor marker.

## History of presentation

A 53-year-old woman, with a history of having been hypertensive for 8 years but displaying no prior record of cardiac illness, came to the hospital with complaints of shortness of breath (class IV) for 1 week. This was aggravated 2 days prior to her visit, associated with orthopnea, a dry cough, and a low-grade intermittent fever. On examination, Pulsus paradoxus and Kussmaul's signs were present.

## Medical history

**Figure 1:**
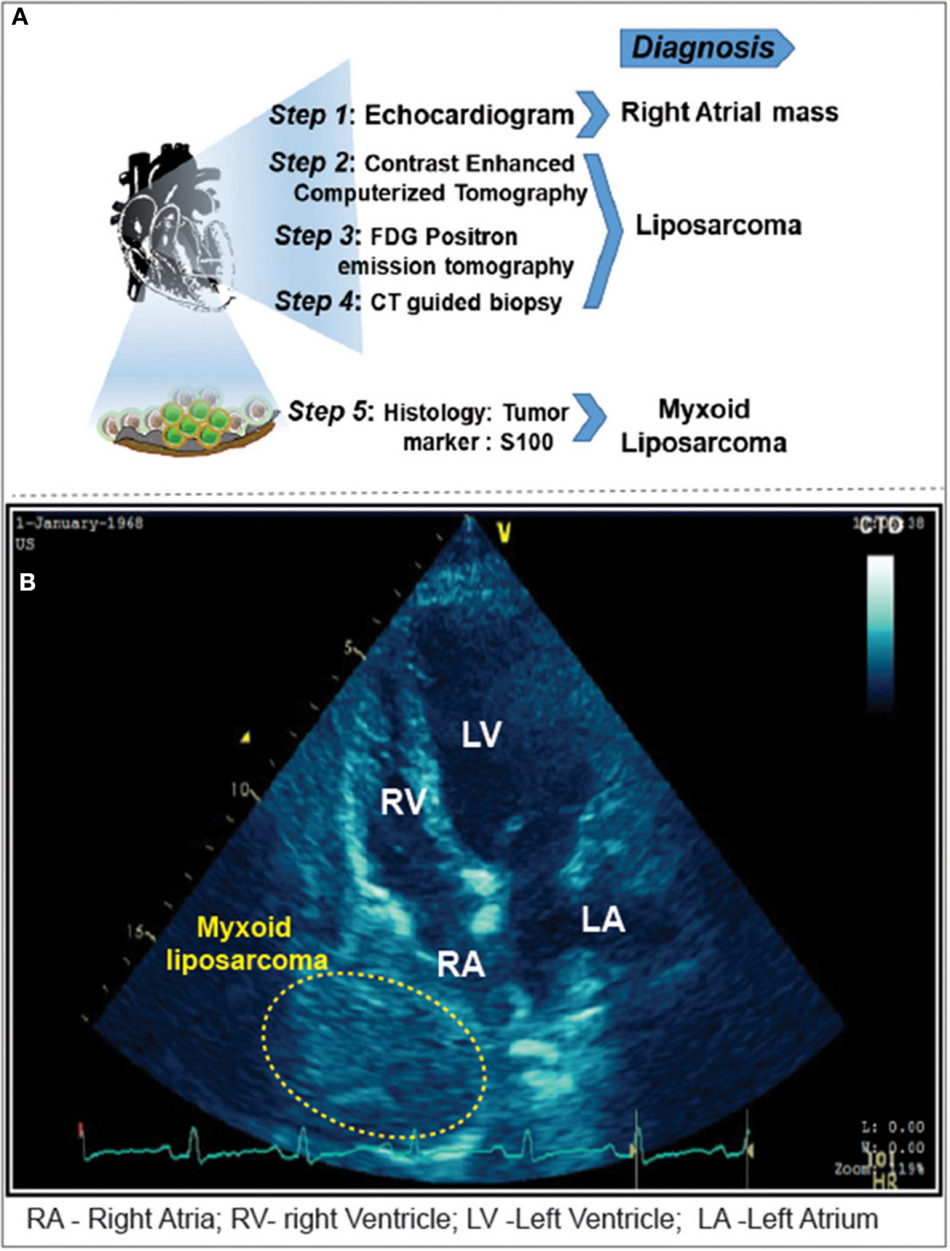
**(A)** Imaging modalities and approaches carried out for the diagnosis of cardiac tamponade. **(B)** Pericardiocentesis followed by 2D-Echocardiogram revealed a 5 x 4 cm sized mass adjacent to the right atrium. Yellow highlighted area locates the myxoid liposarcoma in the right atrium.

The ECG showed sinus tachycardia with non-specific ST-T changes. Moreover, the echocardiogram showed massive pericardial effusion with features suggestive of cardiac tamponade and homogenous mass adjacent to the right atrium (Figures 1A, B). Emergency pericardiocentesis was done, and hemorrhagic pericardial fluid was sent for analysis. The patient became asymptomatic; a chest roentgenogram revealed bilateral minimal pleural effusion. Anti-tuberculous treatment was initiated, given the high suspicion of tuberculosis and mildly raised adenosine deaminase levels in the pericardial fluid sample.

**Figure 2:**
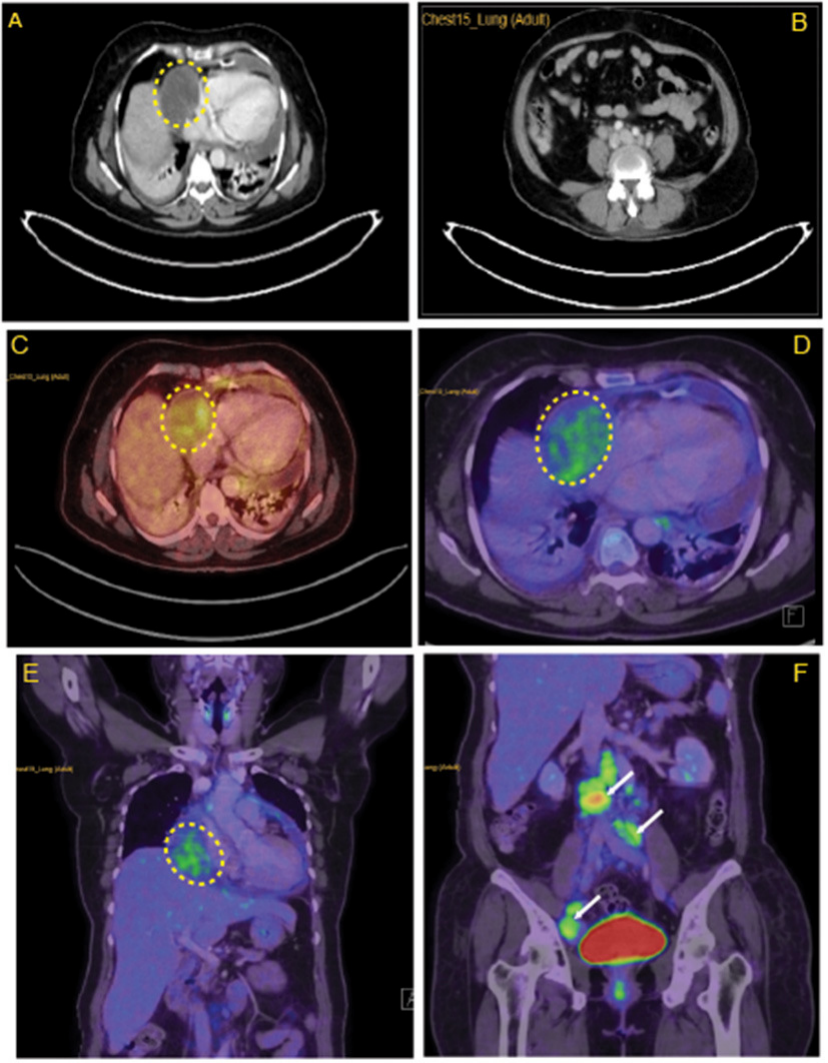
Lymph node characteristics in the right atrium. **(A)** CECT thorax revealing minimally enhancing mass lesion in the right atrial wall. **(B)** Multiple enlarged retroperitoneal lymph nodes. **(C)** Horizontal sections of FDG PET scan revealing mildly FDG avid hypodensity (5.8 × 5 cm, SUV max −3.40) along the lateral wall of the right atrium with mass effect over it. **(D)** Coronal section of FDG PET scan revealing mildly FDG avid mass over the lateral wall of the right atrium. **(E)** Enlarged FDG avid lymph nodes at perivascular, aortocaval, retrocaval, and bilateral common iliac and external iliac levels **(F)**. Yellow highlighted area locates the myxoid liposarcoma in the right atrium. White arrows in **(F)** indicates metastasis in abdomen areas.

CECT thorax (Figures 2A, B) revealed minimally enhancing mass lesion in the right atrial wall with a small intraluminal component projecting into the right atrial chamber, with multiple enlarged retroperitoneal lymph nodes. As it was initially suspected to be lymphoma, a PET CT scan (Figures 2C–F) was performed to rule out metastasis, which showed mildly FDG-avid hypodensity along the lateral wall of the right atrium with mass effect over it.

## Investigations

**Figure 3:**
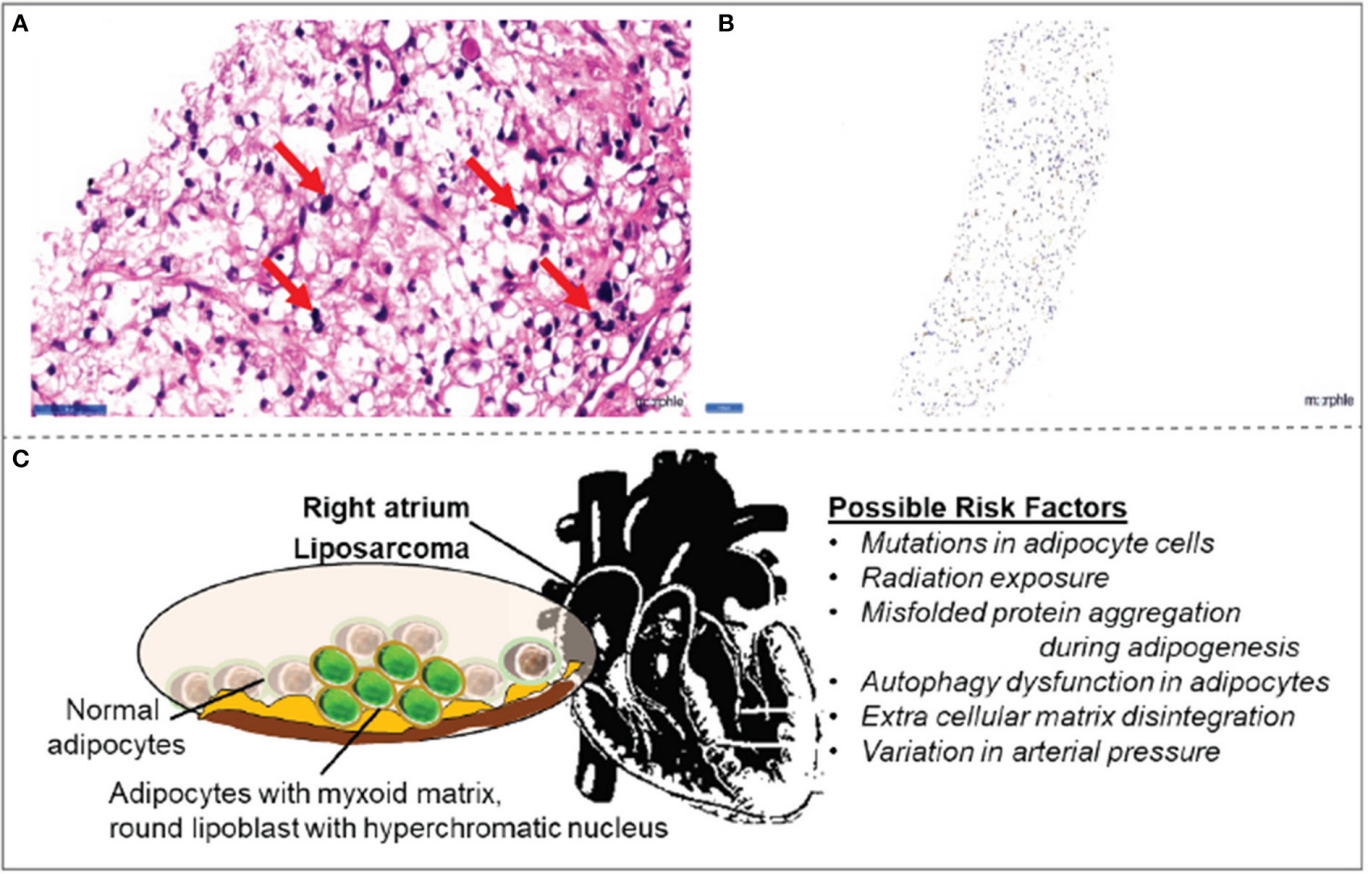
Histopathology observations in right atrial mass **(A)** biopsy collected from right atrial mass demonstrated signet-ring-shaped lipoblasts in mucoid matrix suggestive of myxoid liposarcoma. **(B)** The tumor cells are positive for the S100 tumor marker. **(C)** Schematic illustration of possible risk factors associated with myxoid liposarcomas in the right atrium.

CT-guided biopsy from the right atrial mass was subjected to histopathological examination. The findings revealed the presence of myxoid liposarcoma (Figure 3A). The histological findings were further confirmed by tumor markers Pan CK and S100, denoting that the sample stained positive for S100 (Figures 3A, B).

## Management

Anti-tuberculous treatment was terminated. The case was discussed on the tumor board and a decision to obtain a biopsy from the mesenteric lymph node adjacent to the left iliac artery with laparoscopic assistance was made. However, the patient and her family were not in agreement; hence, she was discharged against medical advice.

## Discussion

Primary cardiac tumors are rare (1–3), found in about 0.001–0.3% of autopsies performed (4–6). Exact incidence of these tumors are unknown or not reported, but 1 in 500 cardiac surgical cases found to have primary cardiac tumors, out of which 75% are benign and about 25% are malignant (7, 8). Of these malignant tumors, around 75% are sarcomas (4, 9). Cardiac sarcoma is classified into right heart sarcomas, left heart sarcomas, and pulmonary artery sarcomas (10). Right heart sarcomas are generally bulkier, exophytic, infiltrate easily, remain asymptomatic, and cause heart failure belatedly and metastasize earlier (11). Therefore, the clinical diagnosis is challenging. The heart is an unusual site for liposarcoma and very few cases of metastatic primary cardiac sarcomas have been reported to date. WHO classification was proposed in 2004 for cardiac tumors (12). This may arise in either of the atria, but also from ventricles and the pericardium. Pericardial involvement may manifest as pericardial effusion or irregular nodules over pericardium (13, 14).

Non-invasive imaging plays a critical role in the diagnosis and surgical interventions of cardiac tamponade (15). Although echocardiography is a simple non-invasive method for cardiac mass evaluation, the area of tissue that could be covered using standard transducers may not be sufficient to assess the extent of the tumor mass (16). In the present case, our multimodal approach involving FDG PET scan followed by CT-guided biopsy facilitated the accurate assessment of size, location, depth of infiltration, and lesion margins of cardiac mass. Histopathology (H&E) of liposarcomas showed a traditional myxoid pattern of the matrix, round lipoblast cells with a hyperchromatic nucleus in the periphery. These cells had scanty cytoplasm, displaced by clear, large lipid droplets appearing as signet-ring shapes. Immunohistochemistry confirmed the S100, a known marker of malignant cells (17, 18). Thus, we recommend the use of an FDG-PET scan followed by histological analysis for the evaluation of size, morphology, tissue characteristics, and clinical correlation of cardiac masses.

Even though there are more innovations in diagnosis (19), the imaging characteristics of myxoid liposarcomas remain inconclusive. Usually, the malignancy tends to arise in atria as a large infiltrating mass; in time, it infiltrates into the ventricles and pericardium. Usually, the pericardium is the most vulnerable site for effusion and tumor nodules (14). While the exact cause of liposarcoma is unclear, variation in arterial pressure, genetic mutations in adipocytes, misfolded protein aggregation during adipogenesis, autophagy dysfunction, and extracellular matrix disintegration are likely to be the molecular mechanisms of the pathology (Figure 3C). In the present case, cardiac tamponade was characterized by pericardial effusion occurring in the right atrium. Of note, the myxoid liposarcoma is positive for S100 tumor markers even in the atrium itself, without any signs of infiltration to ventricles, a very rare occurrence.

Diagnosing myxoid liposarcomas remains a challenge (20, 21). Imaging modalities like echocardiography, cardiac MRI, and CT scans are extremely useful tools for identifying these tumors. FDG-PET scan is the newly evolved tool to diagnose both the tumor and metastasis (22, 23). In this case, we progressed schematically using an echocardiogram for diagnosing cardiac tamponade along with right atrial mass, then imaging with CECT thorax and FDG-PET scan, followed by CT-guided biopsy of the mass. There are limited data and guidelines for the diagnosis and treatment of this rare condition. Total or partial surgical resection may rarely benefit for patients with cardiac tumors. Here, since the sarcoma invaded the whole right atrium of the patient at the time of first hospitalization, a surgical resection or reconstruction was unlikely. However, because the patient was discharged against medical advice, there was no follow-up due to loss of communication.

## Data availability statement

The raw data supporting the conclusions of this article will be made available by the authors, without undue reservation.

## Ethics statement

The studies involving human participants were reviewed and approved by CSP-MED/20/FEB/59/53 DATED 07.03.2020. The patients/participants provided their written informed consent to participate in this study. Written informed consent was obtained from the individual(s) for the publication of any potentially identifiable images or data included in this article.

## Author contributions

MTR and NSR contributed to the intellectual content and revised the manuscript. VK drafted the manuscript. VK and NB were responsible for acquisition and interpretation of the data. SSunn and AR reconstructed the images, prepared the legends, and updated the literature. SSund and PK reviewed the histology data and interpreted the results. JS was responsible for acquisition of CT biopsy. AM performed and interpreted PET scanning. All authors made substantial contribution to the preparation of the manuscript, as well as read and approved the final version of the manuscript.
